# Modeling of free fatty acid dynamics: insulin and nicotinic acid resistance under acute and chronic treatments

**DOI:** 10.1007/s10928-017-9512-6

**Published:** 2017-02-21

**Authors:** Robert Andersson, Tobias Kroon, Joachim Almquist, Mats Jirstrand, Nicholas D. Oakes, Neil D. Evans, Michael J. Chappel, Johan Gabrielsson

**Affiliations:** 1 School of Engineering, University of Warwick, Coventry, UK; 2 Fraunhofer-Chalmers Centre, Chalmers Science Park, Gothenburg, Sweden; 3 iMED CVMD Bioscience Diabetes, AstraZeneca, Gothenburg, Sweden; 4 Division of Pharmacology and Toxicology, Department of Biomedical Sciences and Veterinary Public Health, Swedish University of Agriculture Sciences, Uppsala, Sweden; 5 Systems and Synthetic Biology, Department of Biology and Biological Engineering, Chalmers University of Technology, Gothenburg, Sweden

**Keywords:** Meta-analysis, Turnover models, Nonlinear mixed-effects (NLME), Tolerance, Disease modeling, Dosing regimen

## Abstract

Nicotinic acid (NiAc) is a potent inhibitor of adipose tissue lipolysis. Acute administration results in a rapid reduction of plasma free fatty acid (FFA) concentrations. Sustained NiAc exposure is associated with tolerance development (drug resistance) and complete adaptation (FFA returning to pretreatment levels). We conducted a meta-analysis on a rich pre-clinical data set of the NiAc–FFA interaction to establish the acute and chronic exposure-response relations from a macro perspective. The data were analyzed using a nonlinear mixed-effects framework. We also developed a new turnover model that describes the adaptation seen in plasma FFA concentrations in lean Sprague–Dawley and obese Zucker rats following acute and chronic NiAc exposure. The adaptive mechanisms within the system were described using integral control systems and dynamic efficacies in the traditional $I_{\text{max}}$ model. Insulin was incorporated in parallel with NiAc as the main endogenous co-variate of FFA dynamics. The model captured profound insulin resistance and complete drug resistance in obese rats. The efficacy of NiAc as an inhibitor of FFA release went from 1 to approximately 0 during sustained exposure in obese rats. The potency of NiAc as an inhibitor of insulin and of FFA release was estimated to be 0.338 and 0.436 ${\upmu {\text{M}}}$, respectively, in obese rats. A range of dosing regimens was analyzed and predictions made for optimizing NiAc delivery to minimize FFA exposure. Given the exposure levels of the experiments, the importance of washout periods in-between NiAc infusions was illustrated. The washout periods should be $\sim$2 h longer than the infusions in order to optimize 24 h lowering of FFA in rats. However, the predicted concentration-response relationships suggests that higher AUC reductions might be attained at lower NiAc exposures.

## Introduction

Nicotinic acid (NiAc; or niacin) has long been used to treat dyslipidemia [1, 2]. When given in large doses (1–3 g/day), NiAc improves the plasma lipid profile by reducing total cholesterol, triglycerides, low-density lipoprotein cholesterol, and very-low-density lipoprotein cholesterol, and increasing levels of high-density lipoprotein cholesterol [3]. Moreover, by binding to the G-protein coupled receptor GPR109A, NiAc potently inhibits lipolysis in adipose tissue, leading to decreased plasma free fatty acid (FFA) concentrations [4, 5]. The mechanisms of NiAc-induced antilipolysis have been thoroughly analyzed in previous studies [6–9]. Chronically elevated plasma FFA concentrations are associated with several metabolic diseases, including insulin resistance [10–12]; NiAc-induced FFA lowering is a potential approach to ameliorating these conditions. However, the clinically applied dosing regimens have not been designed to lower FFA; rather, the goal has been to ameliorate dyslipidemia [13].

Although acute administration of NiAc results in rapid reduction in FFA concentrations [2, 14], long-term infusions are associated with tolerance development (drug resistance) and plasma FFA concentrations returning to pre-treatment levels (complete adaptation) [15]. Furthermore, abrupt cessation of the NiAc infusions produces an FFA rebound that overshoots the pre-infusion levels [9, 15]. Numerous studies have sought to quantitatively determine the acute concentration-response relationship between NiAc and FFA [14, 16–23]. The acute NiAc-induced FFA response has been successfully characterized using pharmacokinetic/pharmacodynamic (PK/PD) models, but these models fail to describe the complete return of FFA to pretreatment levels associated with chronic NiAc treatment. Thus, an improved model is required in order to predict optimal treatment regimens, aimed to achieve durable NiAc-induced FFA lowering.

In this study, we sought to further develop the concepts used in previous analyses to develop a more general NiAc-FFA interaction model—applicable to a large set of dosing regimens and NiAc exposure durations. The model was also aimed at quantitatively determining the impact of disease on the FFA-insulin system and to provide predictions for optimal drug delivery. We conducted a meta-analysis on a rich pre-clinical data set of the interaction between NiAc and FFA, as well as insulin, in a nonlinear mixed-effects (NLME) modeling framework. Using various routes and modes of NiAc provocations, we collected concentration-time course data of NiAc (drug kinetics), insulin and FFA (drug-induced dynamics). Experiments were done both in lean Sprague-Dawley and obese Zucker rats—allowing disease impact to be evaluated. Furthermore, by including insulin as a co-variate of the FFA response, we could quantitatively analyze the endogenous antilipolytic effects of insulin [24] under NiAc provocations. Moreover, optimal dosing regimens, consisting of constant rate infusion periods followed by washout periods, were investigated.

## Methods

### Animals

Male Sprague Dawley (lean) and Zucker rats (fa/fa, obese) were purchased from (conscious groups) Harlan Laboratories B.V. (The Netherlands) or (anesthetized groups) Charles River Laboratories (USA). Experimental procedures were approved by the local Ethics Committee for Animal Experimentation (Gothenburg region, Sweden). Rats were housed in an Association for Assessment and Accreditation of Laboratory Animal Care accredited facility with environmental control: 20–22$\,^\circ$C, relative humidity 40–60$\%$, and 12 h light-dark cycle. During acclimatization ($\ge$5 days), animals were housed in groups of 5 with free access to both water and standard rodent chow (R70, Laktamin AB, Stockholm, Sweden).

### Surgical preparations

To prevent potential infections in conjunction with surgery, oral antibiotics were given 1 day before pump/catheter surgery and then once daily for 3 days (sulfamethoxazole and trimethoprim 40 + 8 mg mL$^{-1}$; Bactrim ^®^, 0.2mL /animal, Roche Ltd, Basel, Switzerland). Surgery was performed under isoflurane (Forene^®^, Abbott Scandinavia AB, Solna, Sweden) anesthesia, with body temperature maintained at 37 $^\circ$C. For NiAc/saline administration, a programmable mini pump (iPrecio^®^ SMP200 Micro Infusion Pump, Primetech Corporation, Tokyo, Japan) was implanted subcutaneously, via a dorsal skin incision. To allow blood sampling during the terminal experiment (conscious animals only), a polyurethane catheter (Instech Laboratories Inc, Plymouth Meeting, PA USA) was placed in the right jugular vein via an incision in the neck. In order to maintain its patency up to the acute experiment, the jugular catheter was filled with sterile 45.5% (wt/wt) PVP (polyvinylpyrrolidone, K30, MW $\sim$40,000 Fluka, Sigma-Aldrich, Sweden) dissolved in a sodium-citrate solution (20.6 mmol), sealed and exteriorized at the nape of the neck. Each animal received a post-operative, subcutaneous analgesic injection (buprenorphine, Temgesic^®^, 1.85 $\upmu$g kg$^{-1}$, RB Pharmaceuticals Ltd, Berkshire, GB). Animals were then housed individually and allowed three days of recovery before the start of the pre-programmed pump infusion. Throughout the study, body weight and general health status were monitored and recorded daily.

### Nicotinic acid exposure selection and formulation

A key aspect of the study design was to achieve plateau plasma nicotinic acid (NiAc) concentrations corresponding to therapeutically relevant levels in the rat ($\sim$1 ${\upmu {\text{M}}}$), based on the relationship between plasma NiAc levels and FFA lowering [16]. For intravenous infusions (i.v.), NiAc (pyridine-3-carboxylic acid, Sigma-Aldrich, St. Louis, MO, USA) was dissolved in sterile saline. For subcutaneous (s.c.) infusions, NiAc was dissolved in sterile water and adjusted to physiological pH using sodium hydroxide. Vehicle, for control animals, consisted of sodium chloride solutions at equimolar concentrations. Freshly prepared formulations were loaded into the infusion pump (see below) via a 0.2 $\upmu$m sterile filter (Acrodisc^®^, Pall Corporation, Ann Arbor, MI, USA) just before pump implantation.

### Experimental protocols

#### Conscious animals (NiAc naïve, Cont. NiAc and Inter. NiAc groups)

Both lean and obese animals were divided into 3 dose groups and NiAc was given either acutely (NiAc naïve) or following 5 days of either continuous (Cont. NiAc) or intermittent (Inter. NiAc) administration. Each dose group was matched with corresponding saline infused controls. NiAc infusions were given subcutaneously at 170 nmol min$^{-1}$kg$^{-1}$. The intermittent infusion protocol was programmed as a 12 h on-off cycle (infusion on at 13:00). Following overnight fast, in the morning of the acute experimental day, the jugular catheter was connected to a swivel system to enable blood sampling in unrestrained animals. Jugular catheter patency was maintained by continuous infusion (5 $\upmu$mol min$^{-1}$) of sodium-citrate solution (20.6 mM). After a 3–4 h adaptation period, at $\sim$12:00, the basal phase of the acute experiment commenced with 2–3 blood samples drawn between −60 and −5 min, relative to start of NiAc/saline infusion (note that, in the Cont. NiAc groups, infusion pumps were on throughout this sampling period). Blood samples (16–17/animal) were drawn under an 8 h experimental period. Samples, 30–150 $\upmu$l (with total loss less than 5% of blood volume), were collected in potassium-EDTA tubes, centrifuged and plasma stored at −80 $^\circ$C pending analysis for NiAc, FFA and insulin.

**Fig. 1:**
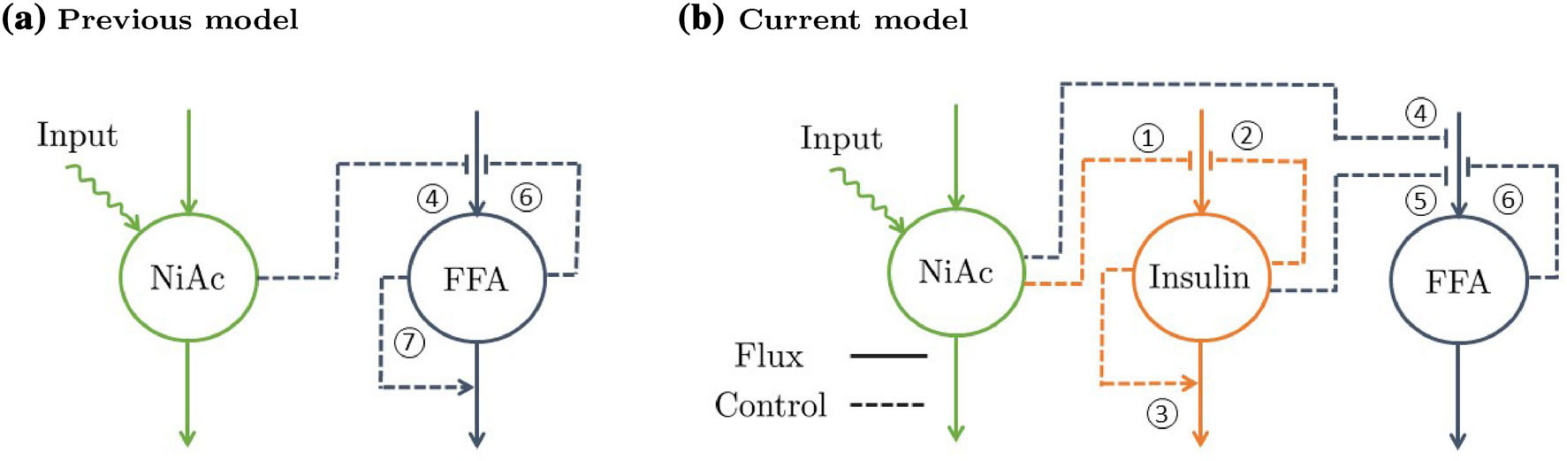
Schematic illustration of how the dependency between NiAc and FFA has been modeled in previous studies (a) and how the dependencies between NiAc, insulin, and FFA were modeled in this study (b). *Solid lines* represent fluxes while *dashed lines* represent control. NiAc inhibits the turnover of insulin (1). Insulin, in turn, has feedback mechanisms that inhibits its turnover (2) and stimulates its fractional turnover (3). Both NiAc (4) and insulin (5) inhibit the turnover of FFA. In this study, FFA has a single feedback mechanism which inhibits its turnover (6), while in previous studies, FFA was modeled using an additional feedback mechanism which stimulates its fractional turnover (7)

#### Anesthetized animals (NiAc Off and NiAc Stp-Dwn 12 h infusion groups)

Before the infusions began, lean and obese rats were fasted for 8 h. On the day of the acute study, at 01:00 (corresponding to time = 0 h), the implanted pre-programmed pump began infusing NiAc at a constant rate of 170 nmol min$^{-1}$ kg$^{-1}$ for 12 h. At 8.5 h animals were anesthetized (Na-thiobutabarbitol, Inactin^®^, 180 mg kg$^{-1}$, i.p., RBI, Natick, MA, USA), underwent a tracheotomy with PE 240 tubing, and breathed spontaneously. One catheter (PE 50 tubing) was placed in the left carotid artery for blood sampling and for recording arterial blood pressure and heart rate. One catheter (PE 10 tubing) was placed in the right external jugular vein to infuse top-up doses of anesthetic. The arterial catheter patency was maintained by continuous infusion of sodium-citrate (20.6 mM in saline, 5 $\upmu$l min$^{-1}$) from shortly after carotid catheterization until the experiment ended. Body temperature was monitored using a rectal thermocouple and maintained at 37.5 $^\circ$C by means of servo controlled external heating. After surgery, animals were allowed a stabilization period of at least 1.5 h and blood sampling began at 11.0 h. At 12.0 h, NiAc infusion was either programmed to switch off (NiAc Off) or to decrease in a step-wise manner, with final switch-off at 15.5 h (NiAc Stp-Dwn). The step-down NiAc infusion rates were 88.9, 58.3, 43.7, 34.0, 24.3, 17.0, and 9.7 nmol min$^{-1}$ kg$^{-1}$. All NiAc protocols were matched with saline-infused controls. Blood samples (18/animal) were drawn during a 6 h experimental period. Samples, 30–150 $\upmu$l (with total loss less than 5% of blood volume), were collected in potassium-EDTA tubes, centrifuged, and plasma was stored at −80 $^\circ$C pending analysis for NiAc, FFA and insulin. All of the experimental groups are summarized in Table 1.

**Table 1:** Summary of experimental protocols—including conscious or anesthetized state, route of administration, duration of experiment, protocol name, and the number of lean and obese rats within each experiment (the number of saline infused controls is given in parenthesis)

	Admin. route	Pre-treat. (h)	Acute exp. (h)	Protocol	Number of rats	
					Lean rats	Obese rats
Conscious animals	Subcutaneous inf.	0	5	NiAc Naïve	7 (2)	7 (5)
		120	5	Cont. NiAc	6 (2)	8 (2)
		120	5	Inter. NiAc	6 (2)	8 (3)
Anaesthetized animals	Intravenous inf.	0	1	NiAc Off 1 h	4 (3)	5 (3)
		0	1	NiAc Stp-Dwn 1 h	5 (2)	5 (2)
	Subcutaneous inf.	0	12	NiAc Off 12 h	5 (2)	4 (2)
		0	12	NiAc Stp-Dwn 12 h	5 (3)	4 (3)

#### Anesthetized animals (NiAc Off and NiAc Stp-Dwn 1 h infusion groups)

After an overnight fast, lean and obese rats were anesthetized and surgically prepared, as described above. They were allowed a stabilization period after surgery of at least 1.5 h. Two basal blood samples were obtained, after which an i.v. NiAc infusion was given at a constant rate (170 nmol min $^{-1}$kg$^{-1}$) for 1.0 h (the start of infusion was taken as time = 0 h). The NiAc infusion was then either switched off (NiAc-Off 1 h) or decreased in a step-wise manner, with final switch-off at 4.5 h (NiAc Stp-Dwn 1 h). The step-down NiAc infusion rates were: 31.1, 20.4, 15.3, 11.9, 8.50, 5.95 and 3.40 nmol min$^{-1}$kg$^{-1}$. All NiAc protocols were matched with saline infused controls. Blood samples (13–18/animal) were drawn during a 6 h experimental period. Samples, 30–150 $\upmu$l (with total loss less than 5% of blood volume), were collected in potassium-EDTA tubes, centrifuged, and plasma was stored at −80 $^\circ$C pending analysis for NiAc, FFA, and insulin.

### Analytical methods

Plasma FFA was analyzed using an enzymatic colorimetric method (Wako Chemicals GmbH, Neuss, Germany). Plasma insulin from obese rats was analyzed with a radioimmunoassay kit (rat insulin RIA kit, Millipore Corporation, St. Charles, Missouri, USA). Plasma insulin concentrations from lean rats were determined using a colorimetric ELISA kit (Ultra Sensitive Rat Insulin ELISA Kit, Crystal Chem INC, Downers Grove, IL, USA). The ELISA was used for lean rats to minimize blood sample volume (only 5 $\upmu$l plasma required vs. $\sim$50 $\upmu$l plasma for RIA). The RIA was used for the obese rats because their high lipid levels in plasma interfere with the ELISA but not the RIA measurement. Due to the hyperinsulinemia in the obese rats only 5 $\upmu$l of plasma was required. For lean-rat plasma (with low lipid levels) the absolute insulin measurements are equivalent for the RIA and ELISA assays, according to an in-house comparison. Plasma NiAc concentrations were analyzed using LC-MS/MS with a hydrophilic interaction liquid chromatography (HILIC) approach, separated on a $50\times$2.1 mm Biobasic AX column, with 5 $\upmu$m particles (Thermo Hypersil-Keystone, Runcorn, Cheshire, UK) as previously described [16].

### Model development

The exposure (PK) and biomarker (PD) models were developed sequentially because of the interaction between the model components; the kinetics of NiAc are assumed to be unaffected by insulin and FFA, whereas NiAc inhibits the release of both insulin and FFA. Furthermore, due to its antilipolytic effect, insulin affects FFA release. The interactions between the three models (NiAc, insulin, and FFA) are illustrated in Fig. 1b, and model interactions of previously published NiAc-FFA models [14, 19, 20, 23] are illustrated in Fig. 1a for comparison. When a sub-model had been estimated, the random effects were fixed to the Empirical Bayes Estimates (EBE) and used as covariates in the subsequent sub-model.

#### Disease modeling and inter-study variability

The PK and PD were significantly different between lean (normal) and obese (diseased) rats and, consequently, these groups were modeled separately. Furthermore, the animal experiments were done under different conditions (separate time periods, anesthetized/conscious animals) which may have provoked different dynamic behaviors. To account for this, inter-study variability was included in the models in the form of fixed-study effects [25].

#### Notation conventions

To improve readability and enable the reader to differentiate between separate sub-model parameters, PD (insulin and FFA) model parameters are labeled with a subscript, indicating to which model they belong. For example, the turnover rate of FFA will be referred to as $k_{\text{inF}}$ and the turnover rate of insulin is $k_{\text{inI}}$ (i.e., F for FFA and I for insulin). Parameters that link NiAc, insulin, and FFA are labeled with both sub-model subscripts (e.g., potency of NiAc as an FFA inhibitor will be called $IC_{\text{50NF}}$, whereby the N is for NiAc and the F is for FFA).

### NiAc exposure model

**Fig. 2:**
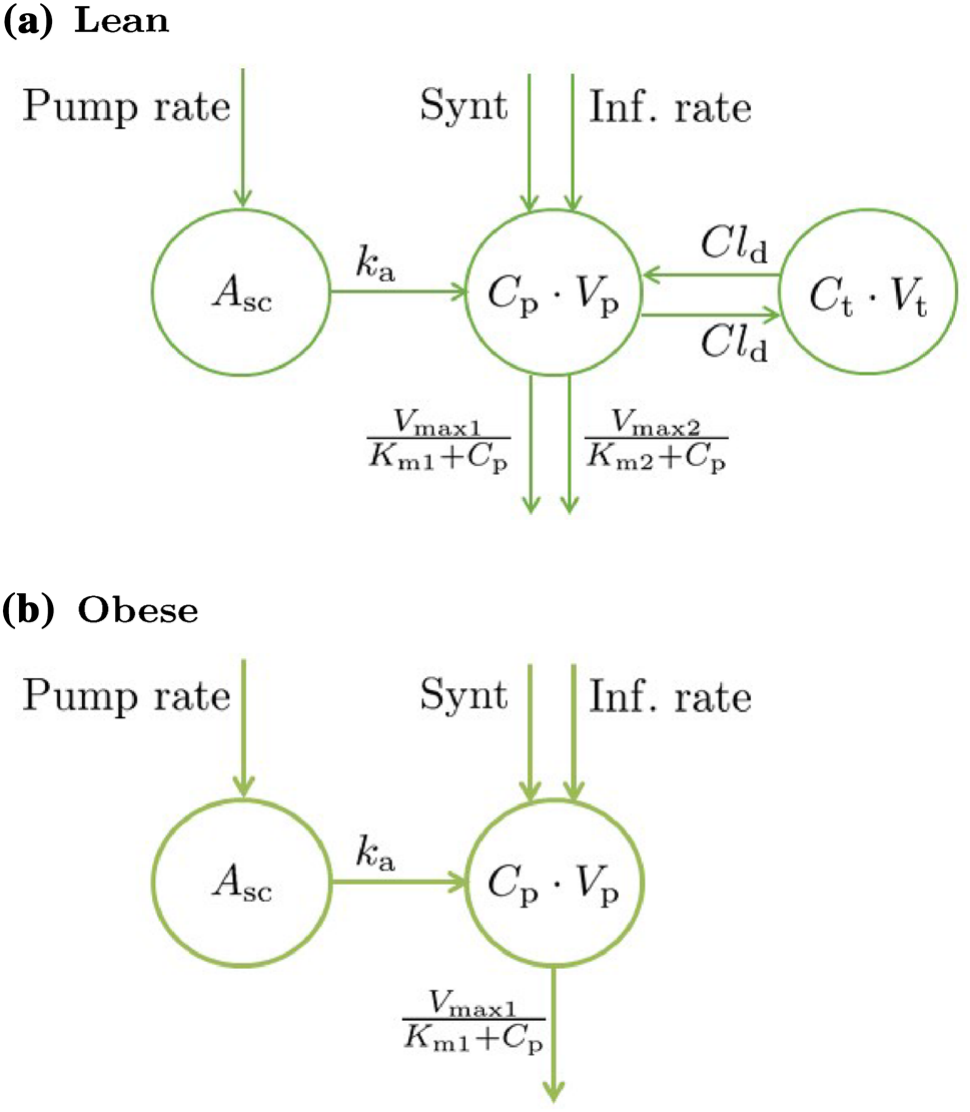
NiAc disposition models for lean Sprague-Dawley (a) and obese Zucker rats (b). NiAc is either infused directly into the central compartment (intravenous administration) or absorbed via a subcutaneous compartment (subcutaneous administration via an implanted mini-pump)

The pharmacokinetic properties of NiAc have been thoroughly characterized in previous studies [14, 16–19, 21–23, 26]. Ahlström et al. [16] introduced a two-compartment disposition model with parallel nonlinear (Michaelis-Menten) elimination for lean Sprague-Dawley rats, and a one-compartmental model with a single nonlinear elimination for obese Zucker rats (a schematic illustration of the PK models is given in Fig. 2).

#### Lean rats

In lean rats, the NiAc disposition is given by

1 $$\begin{aligned} V_{\text{p}}\cdot \frac{{\text d{C_{\text{p}}(t)}}}{\text d{t}}&= {\text{Input}}(t) + {\text{Synt}} - \frac{V_{\text{max1}}\cdot C_{\text{p}}(t)}{K_{\text{m1}}+C_{\text{p}}(t)} \nonumber \\&- \frac{V_{\text{max2}}\cdot C_{\text{p}}(t)}{K_{\text{m2}}+C_{\text{p}}(t)} - Cl_{\text{d}} \cdot C_{\text{p}}(t) \nonumber \\&+ Cl_{\text{d}} \cdot C_{\text{t}}(t), \end{aligned}$$

2 $$\begin{aligned} V_{\text{t}}\cdot \frac{\text d{C_{\text{t}}(t)}}{\text d{t}}&= Cl_{\text{d}} \cdot C_{\text{p}}(t) - Cl_{\text{d}} \cdot C_{\text{t}}(t), \end{aligned}$$

where $C_{\text{p}}(t)$ is the observed NiAc concentration in the central *plasma* compartment and $C_{\text{t}}(t)$ is the concentration in the peripheral *tissue* compartment (derivations of the initial conditions for these compartments are given in Appendix 2), and $V_{\text{p}}$ and $V_{\text{t}}$ are, respectively, the volumes of distribution of the plasma and tissue compartments. The parameters $V_{\text{max1}}$ and $K_{\text{m1}}$ are the maximal elimination rate and the Michaelis constant of the first pathway, and $V_{\text{max2}}$ and $K_{\text{m2}}$ are the maximal elimination rate and the Michaelis constant of the second pathway (low and high affinity pathway, respectively). Furthermore, $Cl_{\text{d}}$ is the inter-compartmental distribution, ${\text{Synt}}$ the endogenous NiAc synthesis, and ${\text{Input}}(t)$ is a time-dependent function determined by the route of administration according to

3 $$\begin{aligned} {\text{Input}}(t) = {\left\{ \begin{array}{ll} {\text{Inf. rate}} \quad &{}{\text{Intravenous infusion}} \\ k_{\text{a}}\cdot A_{\text{sc}}(t) \quad &{}\text{Subcutaneous infusion}, \end{array}\right. } \end{aligned}$$

where ${\text{Inf. rate}}$ is the infusion rate, $A_{\text{sc}}(t)$ is the amount of drug in the subcutaneous compartment, and $k_{\text{a}}$ is the absorption rate from the subcutaneous compartment to plasma. The rate of change of $A_{\text{sc}}(t)$ is given by

4 $$\begin{aligned} \frac{\text d{A_{\text{sc}(t)}}}{\text d{t}}&= {\text{Pump rate}} - k_{\text{a}}\cdot A_{\text{sc}}(t), \end{aligned}$$

with initial condition $A_{\text{sc}}(0)=0$. Here, Pump rate represents the infusion rate from a subcutaneous mini-pump. The mini-pump was surgically implanted seven days before the final acute experiment. During this period, when the pump is not infusing, interstitial tissue fluid may diffuse into the tip of the catheter, diluting the NiAc dosing solution, whilst the solution is leaking into the tissue. Consequently, a concentration gradient may form, resulting in an apparently lower initial infusion rate compared to the pre-programmed setting (particularly pronounced in lean NiAc naïve rats, see Fig. 7a). To capture this, the pump infusion rate is modeled as

5 $$\begin{aligned} {\text{Pump rate}} = {\text{Inf. rate}} \cdot {\text{erf}}\left( \frac{t\cdot \delta }{\sqrt{t_0}} \right) , \end{aligned}$$

where ${\text{Inf. rate}}$ is the programmed infusion rate of the pump, $\delta$ is a lumped diffusion parameter, and $t_0$ is the pump inactivation time (in this case 7 days). Here ${\text{erf}}$ is the error function [27]. The derivation of the Pump rate is given in Appendix 1. Given NiAc’s low molecular weight (123.11 g/mol), bioavailability from the subcutaneous compartment was assumed to be equal to unity.

#### Obese rats

For obese Zucker rats, the NiAc disposition is given by

6 $$\begin{aligned} V_{\text{p}}\cdot \frac{\text d{C_{\text{p}}(t)}}{\text d{t}}&= {\text{Input}}(t) + {\text{Synt}} - \frac{V_{\text{max1}}\cdot C_{\text{p}}(t)}{K_{\text{m1}}+C_{\text{p}}(t)}, \end{aligned}$$

where $C_{\text{p}}(t)$ is the NiAc concentration in the central plasma compartment, $V_{\text{c}}$ the volume of distribution, $V_{\text{max1}}$ the maximal elimination rate, $K_{\text{m1}}$ the Michaelis constant, and ${\text{Synt}}$ the endogenous synthesis. The term ${\text{Input}}(t)$ is the same as for the lean rats (the relations given in Eqs. 3, 4 and 5).

#### Between-subject and residual variability

The modeling was performed in an NLME framework to capture the between-subject variability seen in the exposure-time data. The parameters that varied within the population were $k_{\text{a}}$, $V_{\text{max1}}$, and ${\text{Synt}}$, though ${\text{Synt}}$ varied only in lean rats. These were assumed to be log-normally distributed in order to keep the parameter values positive. However, the five-day continuous infusion group of obese rats did not have exposure data. Consequently, these rats were assumed to behave like the estimated median individual. The residual variability was normally distributed and modeled using a proportional error model.

#### Estimated parameters

Because of sparse sampling, all parameter values could not be estimated from the data. By applying an *a priori* sensitivity analysis [28, 29], we identified the parameters that had the greatest influence on the output. These were then estimated from the data and the remaining parameters were obtained from the literature [23]. The population parameters estimated from the data were $k_{\text{a}}$, $\delta$, and $V_{\text{max1}}$.

### Insulin turnover model

**Fig. 3:**
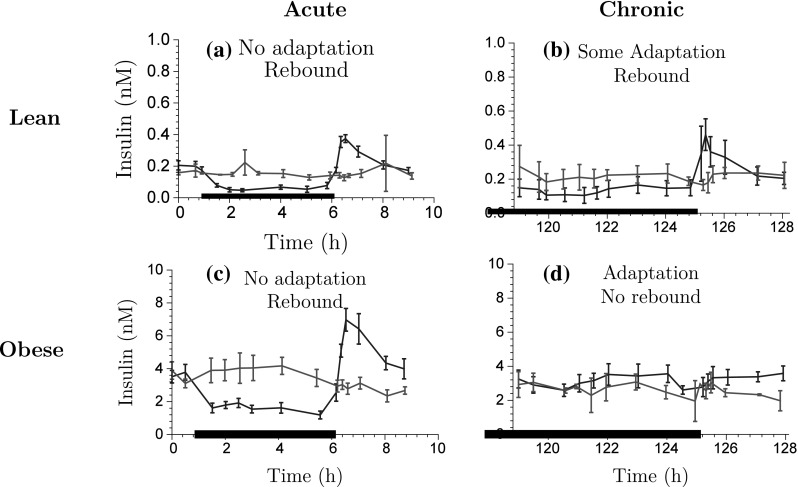
Exploration of insulin-time course data for acute NiAc dosing (a) and (c), and chronic NiAc dosing (continuous infusion) (b) and (d) for lean and obese rats, respectively. The data is presented as the mean response ± the standard error of the mean. The *blue lines* represent the NiAc treated animals, the *red lines* vehicle control group, and the *thick black line* represent the NiAc infusion period (Color figure online)

The primary aim of the insulin model was to establish smooth trajectories that would accurately describe the insulin-time courses under various provocations of NiAc, rather than describe all of the mechanistic aspects of insulin dynamics. To this end, the model structure was kept as simple as possible. The insulin model could subsequently be used to provide an input to the FFA model, enabling a quantitative analysis of the antilipolytic effects of insulin. Given this premise, a phenomenologically based modeling approach was applied. Under the assumption that NiAc perturbs insulin, the characteristics seen in the data were used to establish an insulin model with NiAc as input. The characteristic behavior of the data for acute and long-term NiAc provocations in lean and obese rats is illustrated in Fig. 3. Attributes seen include indirect action, tolerance (drug resistance), rebound, and complete adaptation (insulin levels returning to pre-treatment levels). Data with similar properties as those seen in the acute experiments (Fig. 3a, c) were modeled using turnover equations with moderator feedback control [14, 30]. Furthermore, to capture the different long-term adaptive behaviors with (Fig. 3b), and without (Fig. 3d) rebound, a ’NiAc action compartment’ was included, as well as an integral feedback control. The insulin dynamics are given by

7 $$\begin{aligned} \frac{\text d{I(t)}}{\text d{t}}&= k_{\text{inI}}\cdot R_{\text{I}}(t) \cdot H_{\text{NI}}(C_{\text{p}}(t)) \cdot \frac{M_{\text{0I}}}{M_{\text{1I}}(t)} \nonumber \\&\quad- k_{\text{outI}} \cdot \frac{M_{\text{2I}}(t)}{M_{\text{0I}}} \cdot I(t), \end{aligned}$$

8 $$\begin{aligned} \frac{\text d{M_{\text{1I}}(t)}}{\text d{t}}&= k_{\text{tolI}}\cdot \left( I(t) - M_{\text{1I}}(t) \right) , \end{aligned}$$

9 $$\begin{aligned} \frac{\text d{M_{\text{2I}}(t)}}{\text d{t}}&= k_{\text{tolI}}\cdot \left( M_{\text{1I}}(t) - M_{\text{2I}}(t) \right) , \end{aligned}$$

with initial conditions

10 $$\begin{aligned} I(0) = I_0, \end{aligned}$$

and

11 $$\begin{aligned} M_{\text{1I}}(0)=M_{\text{2I}}(0)=M_{\text{0I}} = I_0, \end{aligned}$$

where *I*(*t*) denotes the observed insulin level, and $M_{\text{1I}}(t)$ and $M_{\text{2I}}(t)$ the first and second moderator compartments, respectively. The parameters $k_{\text{inI}}$ and $k_{\text{outI}}$ are the turnover rate and fractional turnover rate of insulin, respectively, and $k_{\text{tolI}}$ is the fractional turnover rate of the moderators. The regulator compartment $R_{\text{I}}(t)$ is given by

12 $$\begin{aligned} \frac{\text d{R_{\text{I}}(t)}}{\text d{t}} =k_{\text{inRI}} - k_{\text{outRI}}\cdot I(t),\quad R_{\text{I}}(0)=1, \end{aligned}$$

where $k_{\text{inRI}}$ is the turnover rate, $k_{\text{outRI}}$ the fractional turnover rate, and *I*(*t*) the insulin concentration. The regulator compartment is initially at steady-state with

13 $$\begin{aligned} \frac{\text d{R_{\text{I}}(0)}}{\text d{t}} =k_{\text{inRI}}-k_{\text{outRI}}\cdot I_0 = 0 \iff I_0 = \frac{k_{\text{inRI}}}{k_{\text{outRI}}}. \end{aligned}$$

By integrating Eq. 12, the dynamics of $R_{\text{I}}(t)$ can be expressed as

14 $$\begin{aligned} R_{\text{I}}(t)=1+\int _0^t k_{\text{inRI}} - k_{\text{outRI}}\cdot I(\tau )\,{\text{d}}\tau . \end{aligned}$$

Hence, by construction, $R_{\text{I}}(t)$ represents the output of an insulin-driven integral feedback controller [31] with $I_0$ as the set-point and $k_{\text{outRI}}$ as the integral gain parameter ($k_{\text{outRI}}$ will from here on be referred to as the integral gain parameter). The integral feedback controller will ensure that insulin levels return to the baseline $I_0$, despite persistent external effects on insulin turnover and fractional turnover. The inhibitory NiAc function on insulin is given by

15 $$\begin{aligned} H_{\text{NI}}(C_{\text{p}}(t)) = 1 - E_{\text{NI}}(N_{\text{I}}(t))\cdot \frac{ C_{\text{p}}^{n}(t)}{ IC _{\text{50NI}}^{n}+C_{\text{p}}^n(t)}, \end{aligned}$$

where $IC _{\text{50NI}}$ is the potency of NiAc on insulin and *n* the Hill coefficient of the inhibitory function. The term $E_\text{NI}(N_{\text{I}}(t))$ represents the drug efficacy, which is fixed for lean rats and dependent on the concentration in a hypothetical NiAc action compartment, $N_{\text{I}}(t)$, for obese rats, according to

16 $$\begin{aligned} E_{\text{NI}}(N_{\text{I}}(t)) = {\left\{ \begin{array}{ll} I_{\text{maxNI}} &{\text{lean}}\\ I_{\text{maxNI}}\left( 1-\frac{S_{\text{NI}}\cdot N_{\text{I}}^{\gamma }(t)}{ N _{\text{50I}}^\gamma +N_{\text{I}}^\gamma (t)}\right) &{\text{obese}}, \end{array}\right. } \end{aligned}$$

where $I_{\text{maxNI}}$ is the initial efficacy of NiAc on insulin, $N _{\text{50I}}$ the potency of the NiAc action compartment, $S_{\text{NI}}$ the long-term NiAc efficacy loss, and $\gamma$ the corresponding Hill coefficient of the efficacy relation. The dynamics of $N_{\text{I}}$ are in turn given by

17 $$\begin{aligned} \frac{\text d{N_{\text{I}}(t)}}{\text d{t}} = k_{\text{NI}}\cdot (C_{\text{p}}(t)-N_{\text{I}}(t)), \end{aligned}$$

with $N_{\text{I}}(0)=C_{\text{p}}(0)$. Here $k_{\text{NI}}$ is the turnover rate of the NiAc action concentration.

**Fig. 4:**
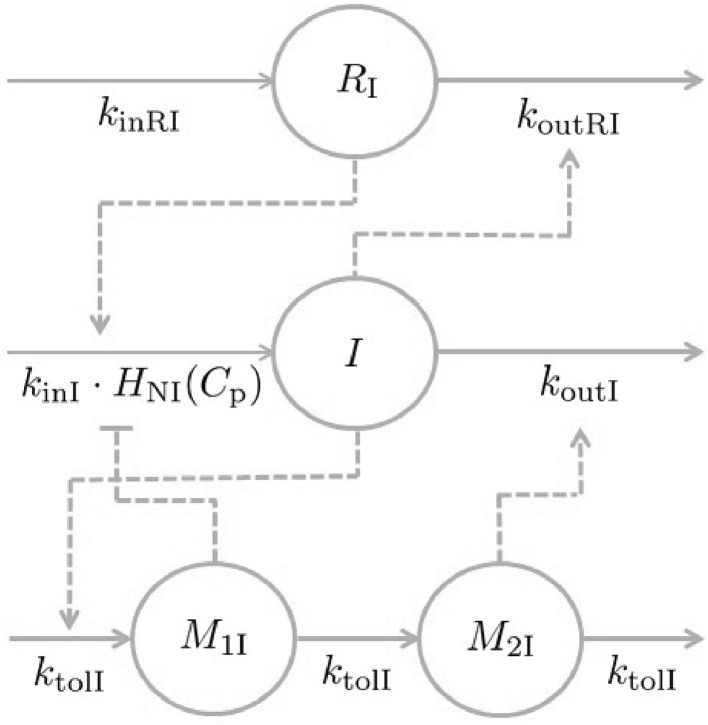
Mechanisms of insulin dynamics. The parameters $k_{\text{inI}}$ and $k_{\text{outI}}$ represent the turnover rate and fractional turnover rate, respectively. The turnover of insulin is inhibited by the NiAc action function $H_{\text{NI}}(C_{\text{p}})$. Tolerance and rebound is captured by the moderator compartments $M_{\text{1I}}$ and $M_{\text{2I}}$, which act on the turnover rate and fractional turnover rate of insulin, respectively. The regulator $R_{\text{I}}$, representing an integral feedback controller, acts on the turnover rate of insulin, in that it strives to maintain insulin baseline, $I_0$, despite persistent external effects on the turnover

The NiAc action compartment is initially at steady-state with the plasma NiAc compartment $C_{\text{p}}$. As infusions begin, and the plasma compartment concentration increases, $N_{\text{I}}(t)$ increases until it reaches the steady-state NiAc concentration $N_{\text{ss}}(t)=C_{\text{pss}}$. With increasing levels in the NiAc action compartment, $E(N_{\text{I}}(t))$ decreases to a minimum of $1-S_{\text{NI}}$ and, consequently, the efficacy of NiAc as an insulin inhibitor is down-regulated. In other words, the system has developed tolerance to the drug. The turnover rate $k_{\text{NI}}$ determines the rate at which tolerance develops. A schematic illustration of the insulin model is given in Fig. 4.

#### Between-subject, inter-study, and residual variability

Individual variations seen in the insulin data were incorporated in the model by allowing the parameters $I_0$, $k_{\text{tolI}}$, and $IC_{\text{50NI}}$ to vary in the population. As in the PK model, these parameters were assumed to be log-normally distributed. The choice of these parameters was guided by an *a priori* sensitivity analysis. Moreover, the parameters $I_0$ and $k_{\text{tolI}}$ varied over study groups according to fixed-study effects on both the mean and individual parameter distributions [25]. In other words, for *S*, the number of groups, the parameter $I_0$ for an individual *j* was modeled as

18 $$\begin{aligned} I_{0j} = (I_{01}\cdot {\text{Study}}_1 + \ldots + I_{0S}\cdot {\text{Study}}_S)\cdot \end{aligned}$$

19 $$\begin{aligned} {\text{exp}}(\eta _1\cdot {\text{Study}}_1 + \ldots + \eta _S\cdot {\text{Study}}_S), \end{aligned}$$

where ${\text{Study}}_k=1$ if individual *j* is in group *k* and 0 otherwise. The residual variability was modeled using an additive model (with normally distributed errors).

### Mechanistic FFA model

**Fig. 5:**
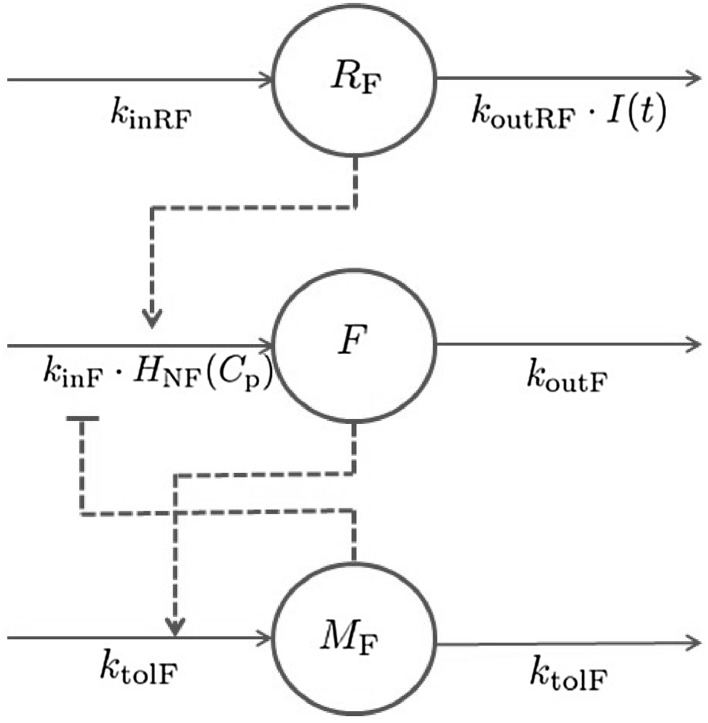
Mechanisms of FFA dynamics. The parameters $k_{\text{inF}}$ and $k_{\text{outF}}$ represent the turnover rate and fractional turnover, respectively. The turnover of FFA is inhibited by the NiAc action function $H_{\text{NF}}(C_{\text{p}})$. Tolerance and rebound are captured by the moderator compartment $M_{\text{F}}$, which acts on the turnover rate of FFA. The regulator compartment *R* acts on the turnover rate of FFA and the fractional turnover rate of *R* is affected by insulin

**Fig. 6:**
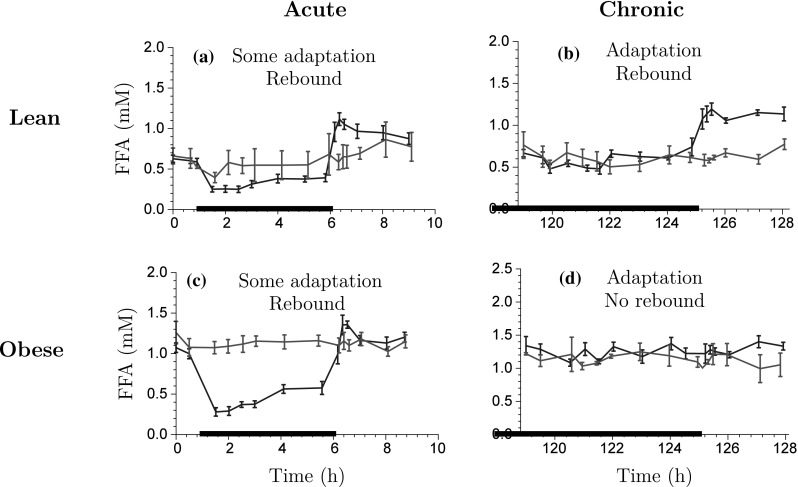
Exploration of FFA-time course data for acute NiAc dosing (a) and (c), and chronic NiAc dosing (continuous infusion) (b) and (d) for lean and obese rats, respectively. The data is presented as the mean response ± the standard error of the mean. The *blue lines* represent the NiAc treated animals, the *red lines* vehicle control group, and the *thick black line* represent the NiAc infusion period (Color figure online)

The model suggested in this study (schematically illustrated in Fig. 5) is founded on preceding approaches [14, 19, 20, 23]; however, insulin has been included as the main endogenous regulator of FFA as insulin provides a homeostatic force on the system—thereby keeping FFA levels in the vicinity of its baseline concentration. Furthermore, the NiAc efficacy is dynamic in that it is decreasing during long-term infusions, which allows for complete systemic adaptation - a feature apparent in the data [32, 33]. The characteristic behavior of the data, for acute and chronic NiAc provocations in lean and obese rats, is illustrated in Fig. 6. Attributes observed include indirect response, tolerance (drug resistance), rebound, and complete adaptation (FFA concentrations returning to pre-treatment levels). The behavior observed in the acute experiment (Fig. 6a, c) has been described by turnover equations with moderator feedback (as described for the insulin system). The long-term behavior, and in particular the adaptations with, and without, rebound, is captured by dynamic NiAc efficacy and an insulin-controlled regulator. The FFA model is given by

20 $$\begin{aligned} \frac{\text d{F(t)}}{\text d{t}}&= k_{\text{inF}} \cdot R(t) \cdot H_{\text{NF}}(C_{\text{p}}(t)) \cdot \frac{M_{\text{0F}}}{M_{\text{F}}(t)} \nonumber \\&- k_{\text{outF}}\cdot F(t), \end{aligned}$$

with initial condition

21 $$\begin{aligned} F(0)=F_0. \end{aligned}$$

Here, *F*(*t*) denotes the observed FFA level, $k_{\text{inF}}$ the turnover rate, and $k_{\text{outF}}$ the fractional turnover rate. The moderator compartment $M_{\text{F}}$ is given by

22 $$\begin{aligned} \frac{\text d{M_{\text{F}}(t)}}{\text d{t}} =k_{\text{tolF}}\cdot \left( F(t)-M_{\text{F}}(t)\right) , \end{aligned}$$

with initial condition

23 $$\begin{aligned} M_{\text{F}}(0)= M_{\text{0F}}=F_0, \end{aligned}$$

where the parameter $k_{\text{tolF}}$ represents the turnover rate of the moderator compartment. The moderator compartment provides a feedback mechanism for the turnover of FFA, that strives to dampen deviations from the baseline response. The regulator compartment $R_{\text{F}}(t)$, that links insulin dynamics to FFA release, is similar to that of the insulin model (Eq. 12) and is given by

24 $$\begin{aligned} \frac{\text d{R(t)}}{\text d{t}} =k_{\text{inRF}} - k_{\text{outRF}}\cdot I(t),\quad R(0)=1, \end{aligned}$$

where $k_{\text{inRF}}$ is the turnover rate, $k_{\text{outRF}}$ the fractional turnover rate, and *I*(*t*) the insulin concentration. As for the insulin regulator, $R_{\text{F}}(t)$ represents the output of an insulin-driven integral controller with $I_0$ as the set-point and $k_{\text{outRF}}$ as the integral gain parameter. The contribution of this integral controller during acute and chronic NiAc treatments in lean and obese rats is illustrated in Fig. 9b. The inhibitory NiAc function on FFA (similar to that for the insulin model, Eq. 15), is given by

25 $$\begin{aligned} H_{\text{NF}}(C_{\text{p}}(t))=1-E_{\text{NF}}(N_{\text{F}}(t))\cdot \frac{ C_{\text{p}}^m(t)}{ IC _{\text{50NF}}^m+C_{\text{p}}^m(t)}, \end{aligned}$$

where $IC _{\text{50NF}}$ is the potency of NiAc as an inhibitor of FFA release and *m* is the Hill coefficient. The drug efficacy is dynamic and changes (down-regulates) during long-term infusions of NiAc. The efficacy is given by

26 $$\begin{aligned} E_{\text{NF}}(N_{\text{F}}(t))= I_\text{maxNF}\cdot \left( 1-\frac{S_{\text{NF}}\cdot N_{\text{F}}^\phi (t)}{ N _{\text{50F}}^\phi +N_{\text{F}}^\phi (t)}\right) , \end{aligned}$$

where $I_{\text{maxNF}}$ is the initial efficacy of NiAc on FFA, $N _{\text{50F}}$ the potency of the NiAc action compartment, $S_{\text{NF}}$ the long-term NiAc efficacy loss, $\phi$ the Hill coefficient, and $N_{\text{F}}(t)$ the concentration in the NiAc action compartment. The dynamics of the NiAc action compartment are in turn described by

27 $$\begin{aligned} \frac{\text d{N_\text{F}(t)}}{\text d{t}} = k_{\text{NF}}\cdot (C_{\text{p}}(t)-N_\text{F}(t)), \end{aligned}$$

with initial condition $N_{\text{F}}(0)=C_{\text{p}}(0)$. Here, the parameter $k_{\text{NF}}$ is the turnover rate of the NiAc action state.

#### Between-subject, inter-study, and residual variability

Random effects were again selected using an *a priori* sensitivity analysis. The parameters that varied in the population were $F_0$, $k_{\text{tolF}}$, and $IC _{\text{50NF}}$ (according to a log-normal distribution). Moreover, inter-study variability was incorporated in the model according to a fixed-study effect (as described for the insulin model). The parameters that varied between experimental groups were $F_0$ and $k_{\text{tolF}}$. The residual variability was modeled using an additive model (with normally distributed errors).

### Numerical analysis

The NLME modelling and simulations and the identifiability analysis were performed using Wolfram Mathematica (Wolfram Research, Inc., Mathematica, Version 10.3, Champaign, IL (2014).

#### Identifiability analysis

All population model structures analyzed in this study were proven to be structurally locally identifiable in a fixed effects setting (identifiability of the population model (fixed effects) implies identifiability of the statistical model (random effects) [34]). The identifiability analysis was performed using the Exact Arithmetic Rank (EAR) approach [35–37]—implemented in the IdentifiabilityAnalysis Wolfram Mathematica package, developed by the Fraunhofer-Chalmers Centre. The EAR algorithm requires that all states and system parameters are rational functions of their arguments. This requirement is not fulfilled in the insulin and FFA systems (for example, the state space variable $C_{\text{p}}$ is raised to the power of *n* in Eq. 15). An illustrative example of how this requirement can be achieved is provided in the Appendix 3.

#### Selection of random effect parameters

An *a priori* sensitivity analysis was used to guide selection of the random parameters [28, 29]. The system output sensitivity, with respect to the parameters, was analyzed and the parameters were ranked accordingly. The parameters with the highest sensitivity, given by the absolute value of the partial derivative of the system output with respect to a specific parameter evaluated at a given point in the parameter space, were considered random in the model.

#### Parameter estimation

Parameter estimates for the NLME models were computed by maximizing the first-order conditional estimation (FOCE) approximation of the population likelihood. This was done using a method developed and implemented in Mathematica 10 (Wolfram Research) at the Fraunhofer-Chalmers Research Centre for Industrial Mathematics (Gothenburg, Sweden) [38], which combines exact gradients of the FOCE likelihood based on the so-called sensitivity equations with the Boyden-Fletcher-Goldfarb-Shanno optimization algorithm [39]. Parameter standard errors were derived using the Hessian of the approximate population likelihood with respect to the parameters, evaluated at the point estimate. The Hessian was computed using finite differences of the exact gradients.

From the steady-state relations in the insulin and FFA models, dependencies were derived which enabled the parameters $k_{\text{inI}}$, $k_{\text{inF}}$, $k_{\text{inRI}}$, and $k_{\text{inRF}}$ to be expressed in terms of other model parameters (derivation given in appendix 2). Consequently, these parameters were redundant and could be replaced in the parameter estimation. Furthermore, some parameters were initially estimated to be very close to their physiological limit (e.g. $I_{\text{maxNI}}=0.9999\approx 1$ for obese rats) and were consequently fixed for numerical stability. Finally, to simplify the parameter estimation, some parameters were fixed (e.g., $S_{\text{NI}}=1$ for obese rats). This is motivated by the complete systemic adaptation apparent in the long-term insulin-time data (obese rats), implying that $S_{\text{NI}}$ must be 1 (The fixed parameters are given in Table 2)). The long-term NiAc efficacy loss for lean rats was initially estimated to be $\approx0$, whereby this part was omitted in the final model.

**Table 2:** Estimates of parameter median values and between-subject variabilities with corresponding relative standard errors (RSE%) for normal Sprague-Dawley rats and obese Zucker rats. Estimates highlighted in blue were taken from the literature (Tapani et al. [23]) while the remaining parameters were estimated in this study

		Normal Sprague-Dawley rats		Obese Zucker rats	
Parameter	Definition	Estimate (RSE$\%$)	BSV$^{a}$ (RSE$\%$)	Estimate (RSE$\%$)	BSV$^{a}$ (RSE$\%$)
Pharmacokinetic model parameters					
$k_{\text{a}}$ (h$^{-1}$)	First order absorption rate	4.27 (13)	80.1 (51)	5.54 (16)	80.2 (47)
$\delta$ (h$^{1/2}$)	Lumped diffusion coeff. catheter	77.4 (15)	–	62.4 (17)	–
$V_{\text{max1}}$ ($\upmu$ mol kg$^{-1}$h$^{-1}$)	Max. elimination - pathway 1	2.64 (12)	93.5 (51)	164 (5.1)	22.4 (13)
$K_{\text{m1}}$ (${\upmu {\text{M}}}$)	Michaelis constant - pathway 1	0.235 (29.2)	–	18.9 (21.5)	–
$V_{\text{max2}}$ ($\upmu$mol kg$^{-1}$h$^{-1}$)	Max. elimination - pathway 2	425 (39.6)	–	–	–
$K_{\text{m2}}$ (${\upmu \text{M}}$)	Michaelis constant - pathway 2	74.5 (43.4)	–	–	–
$V_{\text{p}}$ (L kg$^{-1}$)	Volume of distribution - plasma	0.393 (5.29)	–	0.323 (12.4)	–
$V_{\text{t}}$ (L kg$^{-1}$)	Volume of distribution - tissue	0.172 (35.2)	–	–	–
$Cl_{\text{d}}$ (L kg$^{-1}$h$^{-1}$)	Inter-compartmental distribution	0.0511 (27.8)	–	–	–
${\text{Synt}}$ ($\upmu$mol kg$^{-1}$h$^{-1}$)	Endogenous NiAc synthesis	0.213 (23.3)	66.7 (57)	0.168 (10.1)	95 (110)
$\sigma _{\text{propN}}$	Residual proportional error	0.313 (5.1)	–	0.483 (5.3)	–
Insulin model parameters					
$I_{\text{0}}$ (nM)	Baseline insulin conc.	0.188 (9.7)	49.3 (5.5)	3.26 (12)	10.3 (21)
$k_{\text{outI}}$ (h$^{-1}$)	Fractional turnover rate insulin	6.58 (14)	–	10.8 (17)	–
$I_{\text{maxNI}}$	Efficacy - NiAc on insulin	0.793 (11)	–	1$^b$	–
$IC _{\text{50NI}}$ (${\upmu {\text{M}}}$)	Potency - NiAc on insulin	0.338 (15)	111 (67)	0.175 (27)	190 (160)
*n*	Hill coefficient - NiAc on insulin	3.54 (6.6)	–	0.840 (6.0)	–
$k_{\text{tolI}}$ (h$^{-1}$)	Turnover rate moderator	0.646 (28)	93.9 (20)	0.125 (48)	310 (9.4)
$k_{\text{outRI}}$ (nM$^{-1}$h$^{-1}$)	Integral gain parameter	3.94 (17)	–	0.0612 (27)	–
$k_{\text{NI}}$ (h$^{-1}$)	Turnover rate NiAc action comp.	–	–	0.0242 (35)	–
$N _\text{50I}$ (${\upmu \text{M}}$)	Potency NiAc action compartment	–	–	0.897 (4.9)	–
$\gamma$	Hill coefficient	–	–	18.9 (44)	–
$S_{\text{NI}}$	Long-term NiAc effect loss	–	–	1$^b$	–
$\sigma _{\text{addI}} ({\text{nM}})$	Residual additive error	0.0699 (3.3)	–	0.748 (3.0)	–
Free fatty acid model parameters					
$F_{\text{0}}$ (mM)	Baseline FFA conc.	0.707 (5.0)	17.8 (26)	1.14 (3.1)	0.874 (25)
$k_{\text{outF}}$ (h$^{-1}$)	Fractional turnover rate FFA	428 (140)	–	173 (120)	–
$I_{\text{maxNF}}$	Efficacy - NiAc on FFA	1$^b$	–	1$^b$	–
$IC _\text{50NF}$ (${\upmu \text{M}}$)	Potency - NiAc on FFA	0.436 (12)	41.8 (28)	0.456 (14)	41.8 (26)
*m*	Hill coefficient - NiAc on FFA	1.24 (11)	–	0.731 (9.0)	–
$k_{\text{tolF}}$ (h$^{-1}$)	Turnover rate moderator	1.21 (67)	58.4 (9.6)	0.708 (24)	34.2 (15)
$k_{\text{outRF}}$ (nM$^{-1}$h$^{-1}$)	Integral gain parameter	0.965 (29)	–	0.0165 (38)	–
$k_{\text{NF}}$ (h$^{-1}$)	Turnover rate NiAc action comp.	0.00654 (65)	–	0.0377 (14)	–
$N _\text{50F}$ (${\upmu \text{M}}$)	Potency NiAc action compartment	3.05 (160)	–	0.854 (4.5)	–
$\phi$	Hill coefficient	1$^b$	–	8.83 (33)	–
$S_{\text{NF}}$	Long-term NiAc effect loss	0.807 (190)	–	1$^b$	–
$\sigma _\text{addF} ({\text{mM}})$	Residual additive error	0.130 (3.5)	–	0.135 (3.0)	–

$^{a}$ Between-subject variability expressed in CV%, calculated as $100\times \sqrt{\omega ^2}$.

$^{b}$ Fixed in the estimations.

**Fig. 7:**
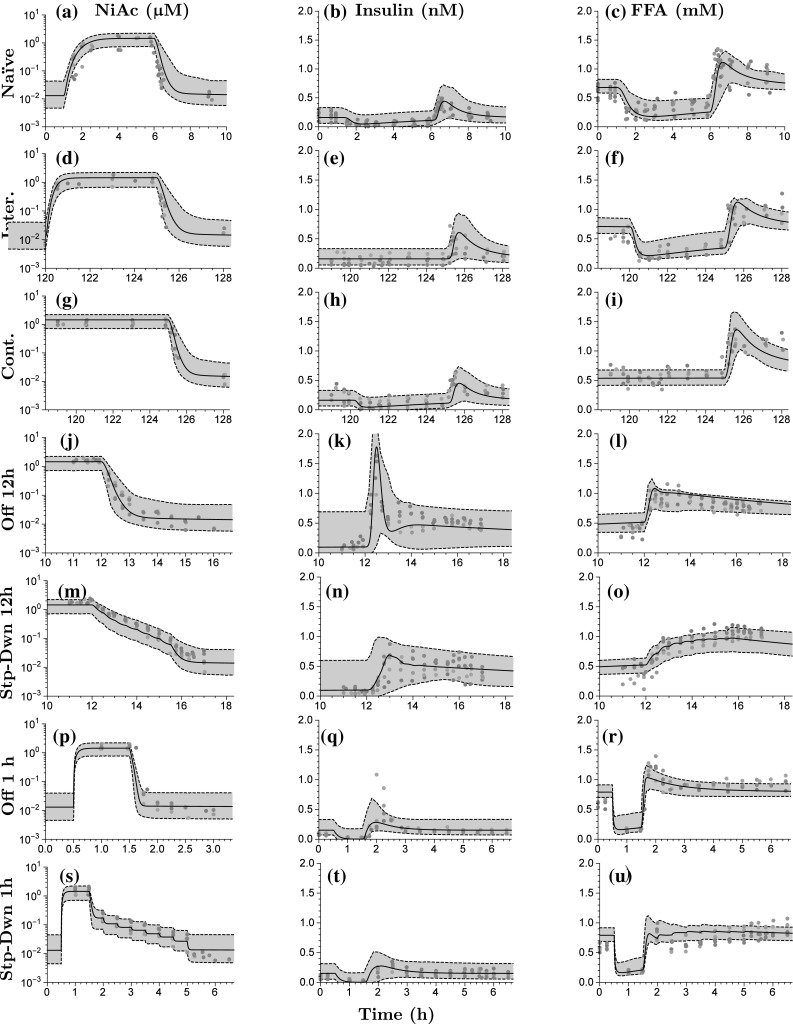
Visual predictive checks for lean Sprague-Dawley rats. The first column shows the PK fit, the second column the insulin, and the third column the FFA. The rows represent the different protocols of NiAc (as described in the Experimental protocols section). The *dots* represent the data, with colors indicating separate individuals, the *black line* the estimated median individual, and the *grey area* the 90% population prediction interval

**Fig. 8:**
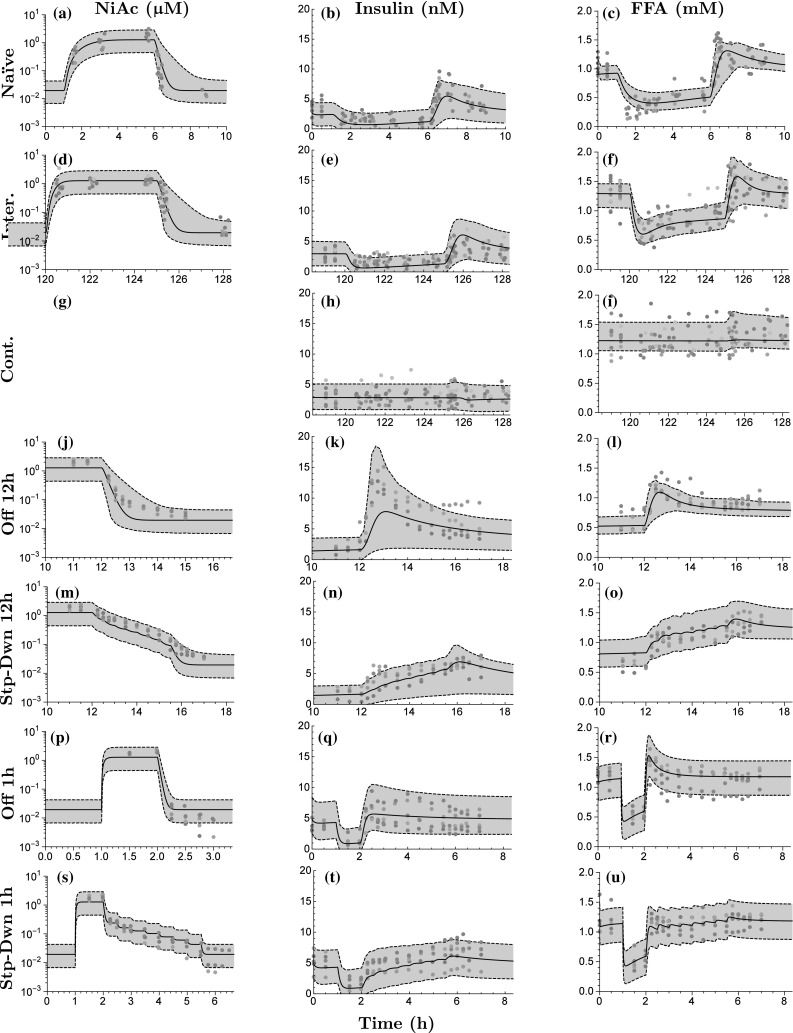
Visual predictive checks for obese Zucker rats. The first column shows the PK fit, the second column the insulin, and the third column the FFA. The rows represent the different protocols of NiAc (as described in the Experimental protocols section). The *dots* represent the data, with colors indicating separate individuals, the *black line* the estimated median individual, and the *grey area* the 90% population prediction interval. No exposure data were available from the Cont. protocol (g).

## Results

The parameter estimation for the three sub-models (NiAc, insulin, and FFA) was performed sequentially, as described in the Model development section. The estimates and between-subject variabilities (expressed in CV%), both with corresponding relative standard errors (RSE%), for normal Sprague-Dawley rats and obese Zucker rats are given in Table 2. Weighted summaries [25] are presented for the parameters that varied between studies. The resulting models were qualitatively evaluated using visual predictive check (VPC) plots [40]; illustrating the data, the model predicted median individual, and 90% Monte Carlo prediction intervals generated from the models [40, 41]. The VPC’s are shown in Fig. 7 for lean Sprague-Dawley rats and in Fig. 8 for obese Zucker rats. The VPC’s are generated from the PK, insulin, and FFA models for all provocations of NiAc.

### Pharmacokinetic model

The pharmacokinetic system reached a steady-state concentration of about ${1}{\upmu {\text{M}}}$ for all protocols both in lean and obese rats (first column in Fig. 7 and first column in Fig. 8). The steady-state was attained faster with intravenous than with subcutaneous administration. When infusions were terminated, the drug was cleared from the system within minutes and the NiAc concentration approached the endogenous level.

The absorption from the subcutaneous compartment had a half-lives of 0.16 and 0.13 h for lean and obese rats, respectively. At steady-state, the elimination of NiAc from the plasma compartment in lean rats was approximately three times faster for the high affinity pathway than the low affinity one. Moreover, the drug elimination rate from the plasma at steady-state was $\sim {20}$ and $\sim {25}{\upmu } {\text{mol}} {\text{kg}}^{-1}{\text{h}}^{-1}$ for lean and obese rats, respectively. The lumped diffusion coefficient was estimated to be 77 and 62 h$^{-1/2}$ for lean and obese rats, respectively, implying that the NiAc dosing solution was diluted during the first $\sim$1.5 h.

### Insulin model

The insulin concentration was suppressed below its baseline value at all provocations of NiAc. The suppression was more pronounced at an early stage of the infusions; at later stages, the insulin concentrations drifted back towards their baselines (cf. Figs. 7b–h or 8b–h). After the infusions were terminated, the insulin concentration rebounded before reaching its baseline value. Rebound was highest in the rats receiving the 12 h Off protocols (Figs. 7k, 8k) and was less pronounced in those receiving step-down protocols (Figs. 7n, t, 8n, t). In obese rats, the insulin concentrations returned to their baselines after long-term infusions of NiAc and did not rebound after the extended infusions were terminated (Fig. 8h).

**Table 3:** Turnover half-lives (expressed in hours) in the insulin and FFA model for lean and obese rats of the biomarkers (insulin or FFA—corresponding rate constant $k_{\text{out}}$), the moderator (rate constant $k_{\text{tol}}$), the integral controller (rate constants $k_{\text{outR}}$), and the NiAc action (rate constant $k_{\text{N}}$)

	Half-lives (h)			
	Lean rats		Obese rats	
Turnover	Insulin	FFA	Insulin	FFA
Biomarker	0.105	0.00162	0.0643	0.00401
Moderator	1.07	0.570	5.53	0.979
Controller	0.176	0.719	11.3	42.0
NiAc action	–	106	28.7	18.4

The median baseline concentrations across groups were 0.233 and 3.51 nM for lean and obese rats, respectively. The estimates of the individual groups ranged between 0.151 and 0.264 nM (inter-study variability of 33%^1^) for lean rats and 2.69–4.82 nM (inter-study variability of 33%$^{1}$) for obese rats. The inter-study variability was not correlated to the anesthetic condition of the rats. The median turnover half-lives of insulin, for the moderator, the integral controller, and the NiAc action level for lean and obese rats are given in Table 3. For lean rats, the efficacy of NiAc on insulin inhibition, $I_{\text{maxNI}}$, was estimated to be 0.793; consequently, NiAc cannot completely inhibit insulin release. The established NiAc exposure was about 1${\upmu {\text{M}}}$ which is approximately three times the NiAc potency related to inhibition of insulin ($IC _{\text{50NI}}=$0.338 ${\upmu {\text{M}}}$). This implies that the inhibitory function was saturated at steady-state. The estimated Hill coefficient *n* indicates a steep NiAc concentration-insulin response relationship at steady-state. Furthermore, for obese rats, the efficacy was fixed to 1 (described in the parameter estimation section) and the corresponding potency was high since the $IC _{\text{50NI}}$ was low in comparison to the NiAc steady-state exposure. However, since the estimated Hill coefficient was $0.84<1$, indicating a gentle NiAc-concentration insulin-response relationship at steady state, the NiAc concentrations never reached levels high enough to saturate the inhibitory function. The estimated $N_{50I}$ of the NiAc action compartment was lower than the steady-state NiAc concentration (0.897<1) and the Hill coefficient of the dynamic efficacy was estimated to be 18.9 (suggesting an all or non-response). This implies that the efficacy was completely down-regulated at the end of the long-term experiments in obese rats, implying no NiAc inhibition on insulin release.

### FFA model

The FFA concentration was suppressed below its baseline value for all provocations of NiAc. Suppression was more pronounced initially during NiAc infusions. FFA concentrations then drifted back towards their baseline values (cf. Figs. 7c–f or 8c–i). After the infusions were terminated, the FFA concentrations rebounded before reaching their baseline values. The step-down protocols resulted in less rebound. The FFA concentrations returned to their baselines during extended exposure of NiAc in lean and obese rats (Figs. 7f, 8i, respectively). However, as the long-term exposure was terminated, rebound occured in lean, but not in obese rats (Figs. 7f, 8i). The median baseline concentrations across groups were 0.707 and 1.14 mM with corresponding ranges of 0.652–0.801 mM (inter-study variability of 11%^2^) in lean and 0.789–1.22 mM (inter-study variability of 20%$^2$) obese rats, respectively. The inter-study variability was not correlated to the anesthetic condition of the rats. The median turnover half-lives of the FFA, the moderator, the integral controller, and the NiAc action level are given in Table 3.

**Fig. 9:**
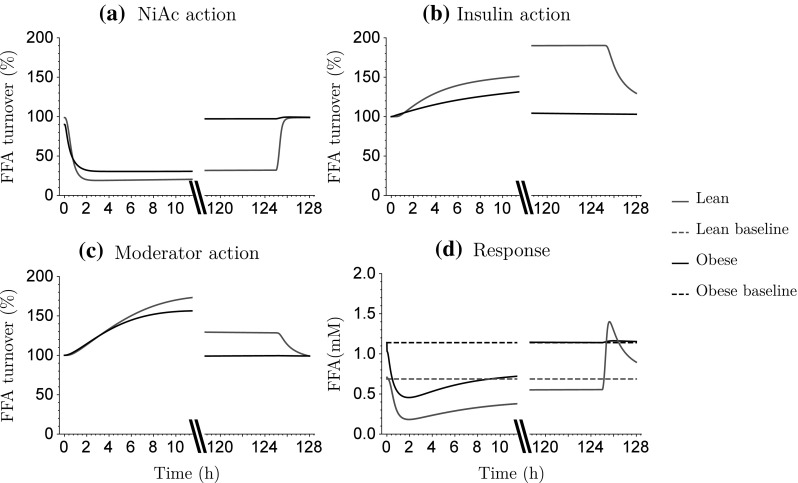
Model simulations of acute and chronic action of NiAc exposure $H_{\text{NF}}$ (a), insulin-driven integral control (b), and moderator feedback (c) on the FFA turnover. Acute and chronic FFA response (d). *Red lines* show lean rats and *black lines* obese rats. The *dashed lines* show the baseline FFA response (Color figure online)

Model simulations of the acute and chronic action of NiAc ($H_{\text{NF}}(C_\text{p}(t))$), the insulin-driven integral controller ($R_{\text{F}}(t)$—also referred to as the insulin-controlled regulator), and the moderator feedback ($M_{\text{0F}}/M_{\text{F}}(t)$) on the FFA turnover are illustrated in Fig. 9 with the corresponding acute and chronic FFA responses. The simulated NiAc concentrations were set to be around 1${\upmu {\text{M}}}$ (corresponding to the experimental exposures). The turnover rate of FFA was initially inhibited about 80% (lean) and 70% (obese) by the NiAc infusion (Fig. 9a). Upon the extended exposure to NiAc (120 h) the inhibitory action on the turnover rate was decreased by approximately 13% due to intrinsic tolerance mechanisms (Eq. 26). In obese rats, the NiAc action vanished completely (Fig. 9a). The insulin-driven controller (Eq. 14) provides a stimulatory action on the FFA turnover rate as insulin concentrations fall below the baseline (Fig.9b). The positive (stimulatory) action increases from 100% (at baseline) to about 200% after extended (120 h) exposure to NiAc in lean rats. The insulin action is totally abolished at equilibrium (120 h) in obese rats (Fig. 9b). The positive impact of the moderator is seen acutely both in lean and obese rats, whereas the moderator action has receded in obese rats at equilibrium (Fig. 9c). In lean rats, the combined inhibitory (NiAc) and stimulatory (insulin, moderator) action on the FFA turnover rate causes a rebound in FFA response when the NiAc infusion is stopped (Fig. 9d). This is not seen in obese rats since all of the NiAc, insulin, and moderator actions are back to baseline at equilibrium (120 h).

The potency of the NiAc action compartment was low for lean rats, since $N _{\text{50F}}>>N(t)$ at equilibrium (120 h), and high for obese rats, since $N _{\text{50F}}<N(t)$ at equilibrium (120 h). Consequently, the loss of efficacy was low in lean rats and high in obese ones. Furthermore, the estimate of Hill coefficient $\phi =8.37$ for obese rats suggests an all-or-none efficacy loss in obese rats.

### Model predictions

The resulting model was used to predict 24 h FFA lowering (AUC$_{24}$) for a range of protocols at steady-state (i.e., after multiple dosing). Here, the lowering over a period *T* is given by

28 $$\begin{aligned} {\text{AUC}}_{\text{T}}=F_0\cdot T - \int _0^T F(\tau )\,{\text{d}}\tau . \end{aligned}$$

**Fig. 10:**
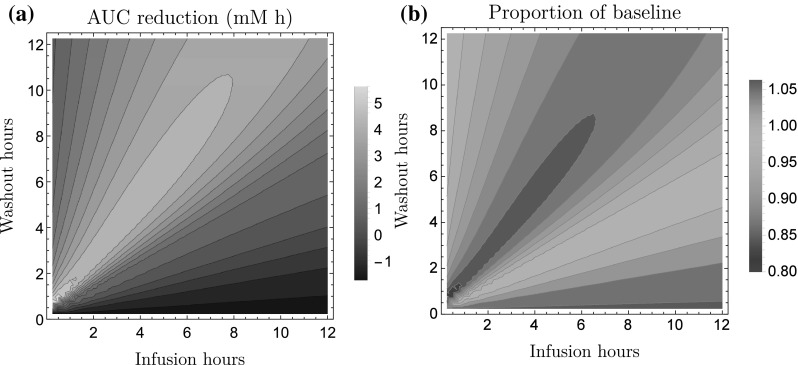
Model predicted reduction in FFA exposure in a median obese rat at steady-state (i.e., after multiple dosing). (a) illustrates the predicted average reduction in 24 h FFA area under the curve and (b) illustrates the proportional reduction, in comparison to the baseline level. The predictions are made for a range of infusion protocols with 0.25–12 h of NiAc exposure followed by 0–12 h washout period. The *x-axis* represents the infusion time and the *y-axis* represents the washout time. The model predicts an optimal infusion regimen of $\sim$2-h longer washout period than the infusion. The maximal AUC reduction is 5.60 mM h

The protocol design consisted of a 0.25–12 h NiAc exposure period followed by a 0–12 h washout period. The NiAc infusions were designed to generate concentrations around the therapeutically relevant level ($\sim$1${\upmu {\text{M}}}$) that was used in the experiments [16]. The predicted AUC$_{24}$ and proportional reduction, in comparison to baseline levels, on a median obese rat are given in Fig. 10. The model predicted an optimal dosing strategy of $\sim$2 h longer washout period than the exposure period and the maximal AUC reduction is 5.60 mM h. These results were consistent when AUC$_{24}$ was predicted for outliers with high/low potencies ($IC _{\text{50NF}}$), baseline responses ($F_0$), and/or moderator turnover rates ($k_{\text{tolF}}$).

**Fig. 11:**
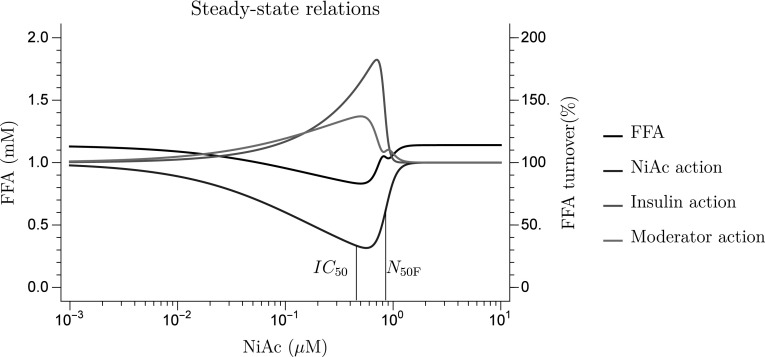
Predicted steady-state concentration-response (left-hand y-axis) and concentration-action (right-hand y-axis) relationships for obese rats. The *black line* represents the FFA response, the *blue line* the inhibitory action of NiAc on FFA turnover, the *red line* the insulin action on FFA turnover, and the *purple line* the moderator action on the FFA turnover. The $IC_{\text{50F}}$ and the $N_{\text{50F}}$ concentrations are given by the *black vertical lines* (Color figure online)

Furthermore, the NiAc-concentration FFA-response relationship at steady-state, for obese rats, is illustrated in Fig. 11, with the corresponding NiAc-, insulin-, and moderator actions. The largest reductions in FFA exposure occurs within a ‘window of opportunity’, with NiAc concentrations between the $IC_{\text{50NF}}$ and the $N_{\text{50F}}$. The predicted AUC$_{24}$ at steady-state, for the optimal NiAc exposure of 0.500${\upmu {\text{M}}}$, was 7.40 mM h.

## Discussion

In this study, we applied a population modeling approach to a unique pre-clinical data set containing FFA-, insulin-, and NiAc-time courses obtained from acute and chronic provocations of NiAc in lean and obese rats. The aim was to identify a general model, from a macro perspective, to be used to predict optimal NiAc exposure profiles and to generate durable chronic dosing regimens.

Recent experimental data from long-term NiAc protocols have illustrated adaptation, with FFA concentration returning to its baseline value [15]. Those findings challenge previous models [18, 23] for long-term dosing predictions.

The PK/PD model applied in this study was derived based on previous models [18, 20, 23], but we added, crucial mechanistic components in order to describe two different kinds of complete adaptation, one seen in lean rats and one in obese. In particular, insulin was included as an endogenous regulator of the turnover of FFA [9]. To this end, a separate insulin model was developed to describe the insulin dynamics for all provocations of NiAc.

### Model characteristics

A key observation of the FFA-time course data in our study is that lean and obese rats both acquire complete adaptation, with FFA levels returning towards baseline at equilibrium (Fig. 6). The post-infusion FFA rebound in lean rats implies a NiAc-sensitive system. However, the rebound was less pronounced than that for the 1–12 h experiments. Consequently, the inhibiting effect of NiAc has been down-regulated during the long-term infusions. For obese rats, the rebound completely vanished, which implies a NiAc-insensitive system. This suggests two separate mechanisms that result in complete adaptation. To describe adaptation with drug effect (seen in lean rats), an insulin-driven integral feedback control was incorporated into the model [39]. Control system techniques have previously been applied in glucose-insulin models in form of PID (proportional-integral-derivative) controllers [42]. The set-point of the insulin-driven integral controller is the insulin baseline and, hence, its action reflects the deviations from baseline insulin concentrations. For the FFA model, the controller represents the antilipolytic effect of insulin [24] via a regulator; as the insulin level increases, the antilipolysis will be more pronounced since the elimination rate of $R_{\text{F}}$ will increase and, consequently, $R_{\text{F}}$’s stimulation of FFA release will be lowered (and vice versa). The traditional $I_{\text{max}}$-equation was modified with a dynamic efficacy function in order to capture the phenomena of NiAc resistance. The dynamic efficacy may represent the down-regulation of the PDE3B gene expression [7].

The impact of the dynamic efficacy and the integral controller (Fig. 9) show that both adaptive actions push FFA concentrations back towards baseline (at equilibrium) despite ongoing NiAc exposure. The insulin-driven controller has less effect in obese as compared to lean rats in spite of 10-fold higher insulin concentrations in the former group. This reflects the insulin resistance of the obese rats [7].

### Model evaluation

The VPC’s (Figs. 7, 8) demonstrate the flexibility of the insulin and FFA models in that response-time courses were captured in both acute and chronic settings. The fractional turnover rate is operating on a significantly shorter time-scale than the feedback and adaptive mechanisms. This resulted in low precision of $k_{\text{outF}}$ (RSE% of 140 and 120, respectively, in lean and obese rats). To achieve higher precision a denser sampling of the FFA time-course is needed during the initial infusion phases. On the other hand, the drug resistance in lean rats was a slower process (half-life of the NiAc action of more than 100 h). This resulted in low precision of the parameters linked to efficacy loss (RSE% of 160 for $k_{\text{NF}}$ and 190 for $S_{\text{NF}}$).

Some estimated median responses of the insulin and FFA models were under- or over-predicted (cf. Fig. 7l or Fig. 7k). This is most likely due to the low number of individuals per trial (5–10), implying that every 4-9th population median will be estimated below or above the trends seen in the individual data [43]. Furthermore, some predicted 90% population spans also under- or over-predicted the response for the insulin and FFA model (cf. Fig. 7l or Fig. 7k). This is most likely due to correlations of the between-subject parameter variabilities, which were not captured because diagonal covariance matrices were used in the FFA model [44]. When sampling from the resulting distributions to generate the VPC’s, non-feasible parameter combinations may occur which render a skewed population [44]. Diagonal covariance matrices were chosen in order to simplify the parameter estimation.

The turnover processes in the insulin and FFA models operate over completely different time scales (Table 3). The general trend in both models is that the moderator and integral controller processes are slower in obese rats. For example, the insulin-driven integral controller has a half-life that is 50 times longer in obese as compared to lean rats. These control processes are tightly controlled in lean animals, and are probably elongated by the tremendous hyperinsulinemia (nearly 10-fold greater pathological concentrations), and corresponding insulin resistance, in obese rats. In the FFA model, turnover of FFA is more than 100 times faster than moderator feedback and integral control. Furthermore, turnover of the NiAc action compartment had a half-life of more than 100 h for lean rats, thus spanning the entire duration of the experiment. The corresponding half-life in obese rats was 18 h. Hence, obese rats reach complete adaptation much faster than lean rats.

Due to the nature of the FFA dynamics, with tolerance development and rebound post-infusions, constructing an optimal dosing protocol is challenging. By selecting an inappropriate dosing regimen, the NiAc provocation can yield an increased FFA exposure in comparison to controls (Fig. 10—the negative AUC area). Given a NiAc exposure of $\sim$1${\upmu {\text{M}}}$ (the exposure that was used in the experiments), there is an optimal strategy whereby washout periods are 2 h longer than infusion periods; this is illustrated by Fig. 10. These strategies were consistent in that they were tested on the median individual and on 90% quantiles (i.e., individuals that had the 5 and 95% quantiles of the parameters that varied in the population: $IC_{\text{50NF}}$, $F_0$, and $k_{\text{tolF}}$). However, rather surprisingly, higher reductions in AUC were attained at constant NiAc exposure (without washout periods) at lower concentrations, where the maximal reduction was 7.40 mM h (in comparison to the maximal reduction of 5.60 mM attained from the exposure/washout protocols).

## Conclusions

Our study presents a novel NiAc-insulin-FFA model that could accurately describe the concentration-response relations seen during acute and chronic NiAc treatment in lean Sprague-Dawley and obese Zucker rats. In particular the model could describe two different types of adaptive changes. This was done by applying an insulin-driven integral controller and a dynamic efficacy in the traditional $I_{\text{max}}$ model. The dynamics of these methods make them suitable for a range of tolerance scenarios. Finally, the model was used to simulate infusion-washout regimens, with a NiAc exposure of 1${\upmu {\text{M}}}$, in order to estimate the 24 h lowering of FFA. Given the targeted exposure, the importance of incorporating washout periods in-between infusions was illustrated. However, the predicted concentration-response relationship suggests that higher reductions in AUC could be obtained by using lower NiAc concentrations. These findings should be experimentally verified in future studies.

## Appendix 1: Diffusion model

The first-order absorption model did not sufficiently describe the full kinetics of the NiAc after subcutaneous administration due to an initial NiAc concentration gradient in the implanted mini-pump catheter. There is a recovery period of 7 days before the experiment begins and the pumps are set into action. During this period, tissue fluid diffuses into the catheter, whilst the drug is leaking into the tissue, which gives rise to a concentration gradient in the catheter. This results in a lower initial infusion rate of NiAc.

The concentration gradient is described by Eq. 5 which is derived by viewing the catheter as a one-dimensional diffusion problem. The one-dimensional fluid equation has the solution

29 $$\begin{aligned} c(x,t)=c_0\cdot {\text{erfc}}\left( \frac{x}{2\sqrt{\hat{\delta }t}}\right) , \end{aligned}$$

where $c_0$ is the concentration at the boundary (where the fluid diffuses from), *x* is the diffusion direction, *t* is the time, $\delta$ the diffusion parameter, and erfc the complementary error function [27]. Given that the intestinal fluids take the form given in Eq. 29, the NiAc concentration in the catheter will, due to symmetry, be scaled by the factor

30 $$\begin{aligned} {\text{erf}}\left( \frac{x}{2\sqrt{\hat{\delta }t}}\right) , \end{aligned}$$

where erf is the error function [27]. Now, assume that the diffusion is negligible after the infusions start and, consequently, only the time between implantation and initiation of the pump, given by $t_0$, affects the concentration gradient. Then the infusion rate will be scaled by the factor in Eq. 29 with $t=t_0$. The direction can be expressed as $x = v\cdot t$ where *v* is the infusion velocity and *t* is the time after the infusion has started. The velocity is fixed in the experiment and can be lumped with the factor $\frac{1}{2\sqrt{\hat{\delta }}}$, as

31 $$\begin{aligned} \delta = \frac{v}{2\sqrt{\hat{\delta }}}, \end{aligned}$$

and the rate is scaled by

32 $$\begin{aligned} {\text{erf}}\left( \frac{t\cdot \delta }{\sqrt{t_0}}\right) \end{aligned}$$

where the parameter $\delta$ is estimated from the data.

## Appendix 2: Steady-state relations

The system given in Eqs. 1 and 2 is at steady-state at $t=0$, and after a couple of minutes, with $\frac{\text d{C_{\text{p}}(t)}}{\text d{t}} =0$. Given this, we can solve for the steady-state concentration $C_{\text{pss}}$($=C_{\text{tss}}$) as

33 $$V_{{\text{c}}} \cdot \frac{{{\text{dC}}_{{{\text{pss}}}} }}{{{\text{dt}}}} = \underbrace {{{\text{input}} + {\text{Synt}}}}_{ = }\alpha - \frac{{V_{{{\text{max1}}}} \cdot C_{{{\text{pss}}}} }}{{K_{{{\text{m1}}}} + C_{{{\text{pss}}}} }} - \frac{{V_{{{\text{max1}}}} \cdot C_{{{\text{pss}}}} }}{{K_{{{\text{m1}}}} + C_{{{\text{pss}}}} }} + \underbrace {{Cl_{{\text{d}}} \cdot C_{{{\text{tss}}}} - Cl_{{\text{d}}} \cdot C_{{{\text{pss}}}} }}_{{ = 0}} = 0,$$

this gives rise to a quadratic equation with the positive real root

34 $$\begin{aligned} C_{\text{pss}}=\frac{-b+\sqrt{b^2-4ac}}{2a}. \end{aligned}$$

Here

35 $$\begin{aligned} a&= \alpha - V_{\text{max1}}-V_{\text{max2}}\end{aligned}$$

36 $$\begin{aligned} b&= \alpha \cdot (K_{\text{m1}}+K_{\text{m2}})-V_{\text{max1}}\cdot K_{\text{m2}}-V_{\text{max2}}\cdot K_{\text{m1}}, \end{aligned}$$

and

37 $$\begin{aligned} c&= \alpha \cdot K_{\text{m1}}\cdot K_{\text{m2}}. \end{aligned}$$

Consequently, the initial steady-state concentration is given by Eqn, 34 with ${\text{Const.}}={\text{Synt}}$ and the steady-state attained after a couple of minutes of infusion is given by Eqn. 34 with ${\text{Const.}}={\text{input}}+{\text{Synt}}$.

Assuming that the system in Eq. 7 is at an initial steady-state with $\frac{\text d{I}}{\text d{t}} =0$ and that the initial plasma concentration of NiAc is close to zero, i.e. $C_{\text{p}}(0)\approx 0$, the inhibitory NiAc function on insulin is $H_{\text{NI}}(C_{\text{p}}(0))\approx 1$ and we obtain

38 $$\begin{aligned} \frac{\text d{I}}{\text d{t}}&= k_{\text{inI}} - k_{\text{outI}}\cdot I_0 = 0 \quad \iff \end{aligned}$$

39 $$\begin{aligned} k_{\text{inI}}&= k_{\text{outI}}\cdot I_0. \end{aligned}$$

Thus, we can eliminate $k_{\text{inI}}$ from the estimation procedure and obtain an estimate for this parameter from Eq. 39.

Assuming that the system in Eq. 20 is at an initial steady-state with $\frac{\text d{F}}{\text d{t}} =0$ and that the initial plasma concentration of NiAc is close to zero, i.e. $C_{\text{p}}(0)\approx 0$, then the inhibitory NiAc function on insulin is $H_{\text{NF}}(C_{\text{p}}(0))\approx 1$ and we obtain

40 $$\begin{aligned} \left. \frac{\text d{F}}{\text d{t}} \right| _{t=0}&= k_{\text{inF}}\left( 1-\underbrace{\frac{I_{\text{maxIF}}\cdot I_0}{ IC _{\text{50IF}}+I_0}}_{\approx 0}\right) - k_{\text{outF}}\cdot F_0 = 0 \quad \iff \end{aligned}$$

41 $$\begin{aligned} k_{\text{inF}}&= k_{\text{outF}}\cdot F_0. \end{aligned}$$

Thus, we can eliminate $k_{\text{inF}}$ from the estimation procedure and obtain an estimate for this parameter from Eq. 41.[45]

## Appendix 3: Identifiability

The insulin system, given by Eqs. 7 to 17, with the NiAc concentration $C_{\text{p}}$ as input and the insulin level *I* as output, does not fulfill the requirement that all states and system parameters must be rational functions of their arguments (for example, the state space variable $C_{\text{p}}(t)$ is raised to the power of *n* in Eq. 15). This issue is overcome by introducing the auxiliary state variable $B(t)=C_{\text{p}}^n(t)$[45] with

42 $$\begin{aligned} \frac{\text d{B(t)}}{\text d{t}} =n \cdot C_{\text{p}}^{n-1}(t)\cdot \frac{\text d{C_{\text{p}}(t)}}{\text d{t}} =n \cdot \frac{B(t)}{C_{\text{p}}(t)}\cdot \frac{\text d{C_{\text{p}}(t)}}{\text d{t}} , \end{aligned}$$

and the parameter $B_0$ as the initial condition

43 $$\begin{aligned} B(0)=B_0\,(=C_{\text{p}}^n(0)). \end{aligned}$$

Furthermore, we introduce $IB _{\text{50}}$ as $IB _{\text{50}}= IC _{\text{50NI}}^n$. Given these transformations, the drug mechanism function in Eq. 15 becomes

44 $$\begin{aligned} H_{\text{NI}}(B(t)) = 1 - E_{\text{NI}}(N(t))\frac{B(t)}{ IB _{50}+B(t)}, \end{aligned}$$

and the augmented insulin system, with Eq. 42 to 44 with parameters $B_0$ and $IB _{50}$ included, will fulfil the requirements of the EAR algorithm (this augmented system is equivalent to the original one). Now, explicit relations such as $B_0=C_{\text{p}}^n(0))$ can not be included in the algorithm. This leaves $B_0$ structurally locally unidentifiable and, consequently, $IB _{50}$ structurally locally unidentifiable. However, all other parameters are structurally locally identifiable and the degree of freedom of the system, given by the algorithm, is 1. Given that we can identify *n* and that we know the initial input $C_{\text{p}}(0)$, we can identify $B_0$ from $B_0=C_{\text{p}}^n(0))$. Hence, we can also identify $IB _{50}$ (since the degree of freedom was 1) and the system is structurally locally identifiable.

## Footnotes

1 Expressed in CV%

2 Expressed in CV%
